# Activation of Peroxisome Proliferator-Activated Receptor-Gamma by Glitazones Reduces the Expression and Release of Monocyte Chemoattractant Protein-1 in Human Mesothelial Cells

**DOI:** 10.1155/2012/217696

**Published:** 2012-02-07

**Authors:** Matthias Sauter, Kathrin Kastenmüller, Franziska Belling, Markus Wörnle, Roland Ladurner, Thomas Mussack, Thomas Sitter

**Affiliations:** ^1^Medizinische Poliklinik-Innenstadt, Klinikum der Universitaet Muenchen, Pettenkoferstr. 8a, 80336 Muenchen, Germany; ^2^Chirurgische Klinik-Innenstadt, Klinikum der Universitaet Muenchen, Nußbaumstr. 20, 80336 Muenchen, Germany

## Abstract

Human peritoneal mesothelial cells (MC) play an important role in inflammatory processes of the peritoneal cavity by producing various cytokines and chemokines, such as monocyte chemoattractant protein-1 (MCP-1). The present study was designed to assess the effect of the peroxisome proliferator-activated receptor-gamma- (PPAR*γ*-) activator rosiglitazone on the mesothelial MCP-1 expression and release. Primary cultures of MC were obtained from omental tissue. MCP-1 antigen concentrations were measured in the cell supernatant by ELISA and MCP-1 mRNA levels by real-time polymerase chain reaction. The presence of PPAR*γ* on MC was assayed in a Western Blot analysis. MC constitutively express PPAR*γ*. Activation of this receptor via rosiglitazone (0,1–10 *μ*mol/L) resulted in significantly reduced amounts of mesothelial MCP-1 release as well as MCP-1 mRNA. The use of the PPAR*γ* inhibitor GW-9662 could completely prevent the rosiglitazone effects. Rosiglitazone was also effective in reducing TNF*α*-induced enhanced secretion of MCP-1. Our findings indicate that glitazones are effective in reducing constitutive and TNF*α*-stimulated mesothelial MCP-1 mRNA expression and release.

## 1. Introduction

Mesothelial cells (MC), which form the innermost monolayer of the peritoneal cavity, are critical in morphological and functional alterations of the peritoneal membrane in patients who undergo peritoneal dialysis (PD). They are a major source of intraperitoneal monocyte chemoattractant protein-1 (MCP-1) which is a chemokine that has been reported to play a key role in the recruitment of monocytes toward the peritoneal cavity [1]. Monocytes in turn contribute to peritoneal fibrosis by producing various cytokines and growth factors [2] that induce cell proliferation and extracellular matrix production in mesothelial cells and fibroblasts [3, 4]. 

Thiazolidinediones (TZD) are a novel group of antidiabetic agents that act via the activation of peroxisome proliferator-activated receptor-gamma (PPAR-*γ*), a nuclear hormone receptor. PPAR-*γ* regulates a variety of metabolic pathways as a transcription factor [5]. Therefore, TZD like rosiglitazone are not only capable of increasing the insulin sensitivity in peripheral organs (as e.g., adipose tissue) and thus lowering blood glucose levels in diabetic patients; they also possess anti-inflammatory capacities in certain circumstances as they decrease the expression of inflammatory proteins like iNOS and MMP9 in macrophages [6, 7]. A reduced expression of MCP-1 due to TZD treatment has been shown for diverse cell types as, for example, lung epithelial [8], endothelial [9], and mesangial cells [10]. In this context, the present study was designed to investigate the presence of PPAR-*γ* on human peritoneal MC and to characterize the effect of PPAR-*γ* activation by TZD on mesothelial MCP-1 mRNA transcription and release.

## 2. Materials and Methods

### 2.1. Materials

Medium M199 and newborn calf serum were obtained from Gibco BRL (Eggenstein, Germany); tissue-culture plates came from Costar (Cambridge, Massachusetts, USA). Human serum was prepared from freshly collected blood of healthy donors and stored at –20°C. Fibronectin from human serum, trypsin, and TNF*α* were purchased from Boehringer (Mannheim, Germany), collagenase type II from Worthington (Freehold, NY, USA).

Monoclonal antibodies against cytokeratins 8 and 18 as well as vimentin were a gift from Dr. G. van Muijen (University of Nijmegen, The Netherlands). Antibody against PPAR-*γ* was from Santa Cruz Biotechnology (Santa Cruz, California, USA). MTT was from Sigma-Aldrich (St. Louis, MO, USA). Rosiglitazone was from Molekula (Nienburg/Weser, Germany), and GW-9662 was purchased from Cayman chemical (Ann Arbor, Michigan, USA).

### 2.2. Cell Culture Experiments

MC were isolated from the omental tissue of consenting patients undergoing elective surgery, as described previously [11]. The patients were free from peritonitis or peritoneal carcinosis. Cells were grown in fibronectin-coated dishes in M199 medium supplemented with 25 mmol/L HEPES (pH 7.3), 2 mmol/L glutamine, 10% (v/v) human serum and 10% (v/v) newborn calf serum (heat inactivated), penicillin (100 IU/mL), and streptomycin (100 *μ*g/mL) at 37°C under 5% CO^2^/95% air atmosphere. The medium was replaced every 2 to 3 days. Subcultures were obtained by trypsin/EDTA treatment at a split ratio of 1 : 3. The cells were purely MC, as assessed by their uniform cobblestone appearance at confluence, by the absence of von Willebrand factor, and by their uniform positive staining for cytokeratins 8 and 18 as well as for vimentin. For the experiments, confluent cultures were used at the second or third passage, and cells were refed with 2% human serum 24 hours before the experiment. Conditioned media were obtained by incubating cells in 2 cm^2^ dishes at 37°C with 0.5 mL serum-free M199 containing the appropriate concentration of the test compound. Serum-free medium M199 served as a control. In coincubation experiments with TNF*α*, cells were preincubated for 24 hours with rosiglitazone, and then TNF*α* was added. In experiments using a PPAR-*γ* blocker, cells were preincubated with GW-9662 for 24 hours. Supernatants were centrifuged 5 minutes at 2000 ×g to remove cells and cellular debris, and the samples were frozen at –20°C until use. All experiments were done with cells from 3 to 6 individual donors and were measured in triplicate.

### 2.3. Western Blot Analysis

Cultured human mesothelial cells were harvested with lysis buffer (50 mM Tris-HCl pH7.4, 1% Nonidet P-40, 0.25% sodium deoxycholate, 150 mM NaCl, 1 mM EGTA, and 1 mM Na3VO4, Complete Protease Inhibitor Cocktail (Roche, Mannheim, Germany)). Extracted proteins were boiled in loading buffer for 30 min, resolved by 8% SDS-PAGE under reducing conditions, and transferred to an Immobilon-P membrane (Millipore, Eschborn, Germany). The membrane was blocked with 3% skim milk, incubated in a 1 : 1000 dilution of a rabbit polyclonal IgG-antibody against human PPAR*γ*-1 (SC-7196, Santa Cruz Biotechnology, Heidelberg, Germany) over night, and rinsed with PBS containing 0.1% Tween 20. Immune complexes were visualized using enhanced chemoluminescence (ECL, Amersham Biosciences, Freiburg, Germany). Human breast carcinoma cell lysate protein extract served as positive control for PPAR*γ*-1 detection.

### 2.4. MTT Assay

Human mesothelial cells (30 × 10^3^/100 *μ*L medium) were cultured in 96-well microtiter plates for 24 h under standard conditions to yield firmly attached and stably growing cells. After discarding supernatants, 50 *μ*L of medium M199 containing rosiglitazone in concentrations of 1 and 10 *μ*g/L or medium M199 as a control was added to the cells and incubated for 48 h. Then 50 *μ*L of a 1 mg/mL solution of (3,5-Dimethylthiazol-2-yl]-2,5-diphenyl-tetrazolium bromide) MTT (SIGMA-ALDRICH, Taufkirchen, Germany no. M2128) was added. After 3 h incubation at 37°C, formazan crystals were dissolved by addition of 100 *μ*L isopropanol and 0.04 N HCl. Absorbance was then measured at 590 nm using a GENios plus TECAN ELISA reader. For each experiment at least 6 wells were analyzed per experimental condition.

### 2.5. MCP-1 Assay

MCP-1 antigen levels [pg/10^5^ cells] were measured by Quantikine human MCP-1 immunoassay from R&D Systems (Minneapolis, MN, USA). Diluted aliquots of the cell supernatants were assayed without prior purification.

### 2.6. RNA Isolation and Real-Time Quantitative RT-PCR

Total RNA was extracted from cells using silica gel columns (RNeasy, Qiagen, Hilden, Germany). 2 *μ*g of isolated total RNA underwent random hexamer-primed reverse transcription for one hour at 42°C using a modified Molony murine leukaemia virus (MMLV) reverse transcriptase (Superscript, Life Technologies, Karlsruhe, Germany). Real-time quantitative reverse transcription-polymerase chain reaction (RT-PCR) was performed on a Taq-Man ABI 7700 Sequence Detection System (PE Biosystems, Weiterstadt, Germany) using heat-activated TaqDNA polymerase (Amplitaq Gold, Applied Biosystems, Darmstadt, Germany). Thermal cycler conditions contained holds for two minutes at 50°C and ten minutes at 95°C, followed by 40 cycles of 15 seconds at 95°C and one minute at 60°C. Message expression was calculated following the ΔΔCt procedure [12]. Glyceraldehyde-3-phosphate dehydrogenase (GAPDH) and 18S ribosomal RNA (rRNA) served as the reference housekeeping gene. Controls consisting of H_2_O or samples that were not reverse transcribed were negative for the target and reference. Sequences with following gene bank accession numbers served for the design of the predeveloped Taq Man assay reagents (PDAR) or primers and probe, purchased from Applied Biosystems (Darmstadt, Germany): X14768 (human MCP-1/CCL2) M33197 (human GAPDH) and X03205.1 (human18s-rRNA).

### 2.7. Statistical Analysis of the Data

Data are given as mean ± SD. Statistical analysis was performed using the ANOVA analysis. A *P* value < 0.05 was considered to indicate statistically differences.

## 3. Results

### 3.1. Detection of Constitutive PPAR-*γ* Expression in MC

**Figure 1:**
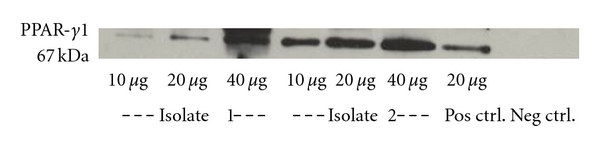
Western Blot analysis of PPAR*γ*-1 in two different isolates o unstimulated mesothelial cells. Total protein was extracted from unstimulated peritoneal mesothelial cells or human breast carcinoma cells (positive control) and were analysed using the Western Blot technique. The amount of total protein loaded is indicated. Pos. ctrl: positive control; neg. ctrl: negative control.

We evaluated the presence of PPAR-*γ*1 on human MC via Western Blot. Analysis of the extracted total protein of unstimulated MC showed a single band at 67 kDa as has been described for PPAR-*γ*1 and was comparable to the recommended positive control (protein from human breast carcinoma cells) (Figure 1).

### 3.2. Effect of Rosiglitazone on the Secretion of MCP-1 in Unstimulated MC

**Figure 2:**
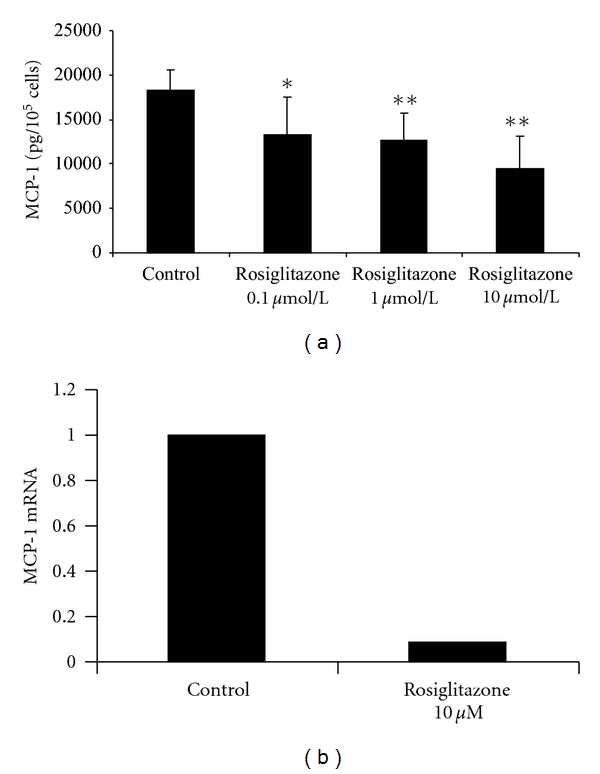
(a) Effect of rosiglitazone on mesothelial MCP-1 release. MC were stimulated with rosiglitazone in the given concentrations for 48 hours. MCP-1 antigen was measured in the cell culture supernatants using ELISA technique. *indicates a *P* value < 0.05; **indicates a *P* value < 0.01 (*n* = 6). (b) Effect of rosiglitazone on mesothelial MCP-1 mRNA levels. MC were incubated with rosiglitazone 10 *μ*M for 4 hours. Total RNA was extracted and analysed via RT-PCR. MCP-1 mRNA levels were adjusted to the housekeeper rRNA and are expressed as relative to the control. The figure is a representative of three independent experiments.

**Figure 3:**
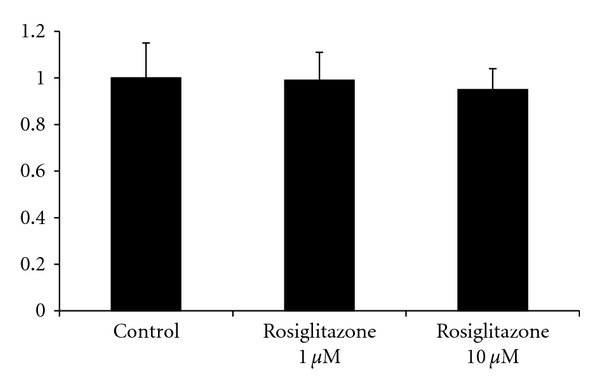
Effect of rosiglitazone on mesothelial cell viability and proliferation. MC were stimulated with rosiglitazone in the given concentrations for 48 hours, and then a MTT test was performed as described in the Methods section. Results are described in relation to the control.

Confluent unstimulated MC were incubated with increasing concentrations of rosiglitazone (0.1–10 *μ*mol/L) for 48 hours. This resulted in a concentration-dependent decrease of MCP-1 protein levels in the cell culture supernatants. Rosiglitazone treatment reached statistical significancy at each employed concentration: A concentration of 0.1 *μ*mol/L reduced the MCP-1 level to 13200 pg/10^5^ cells versus control 18200 pg/10^5^ cells (*P* = 0.013) whereas a concentration of 1 *μ*mol/L reduced MCP-1 levels to 12700 pg/10^5^ cells (*P* = 0.001) and a concentration of 10 *μ*mol/L to 9400 pg/10^5^ cells (*P* < 0.001) (Figure 2(a)). Rosiglitazone treatment in the shown concentrations did not effect cell viability as assessed in trypan blue staining or cell proliferation as assessed in an MTT assay (Figure 3).

### 3.3. Effect of Rosiglitazone on the MCP-1 mRNA Levels in Unstimulated MC

The incubation of MC with 10 *μ*mol/L rosiglitazone resulted in a marked decrease of MCP-1 mRNA after a 4 hours' dwell time (0.09 versus 1) (Figure 2(b)). A longer incubation time (8 hours) and the use of another housekeeping gene (GAPDH) resulted in comparable findings (data not shown).

### 3.4. Impact of PPAR-*γ* Inhibition

**Figure 4:**
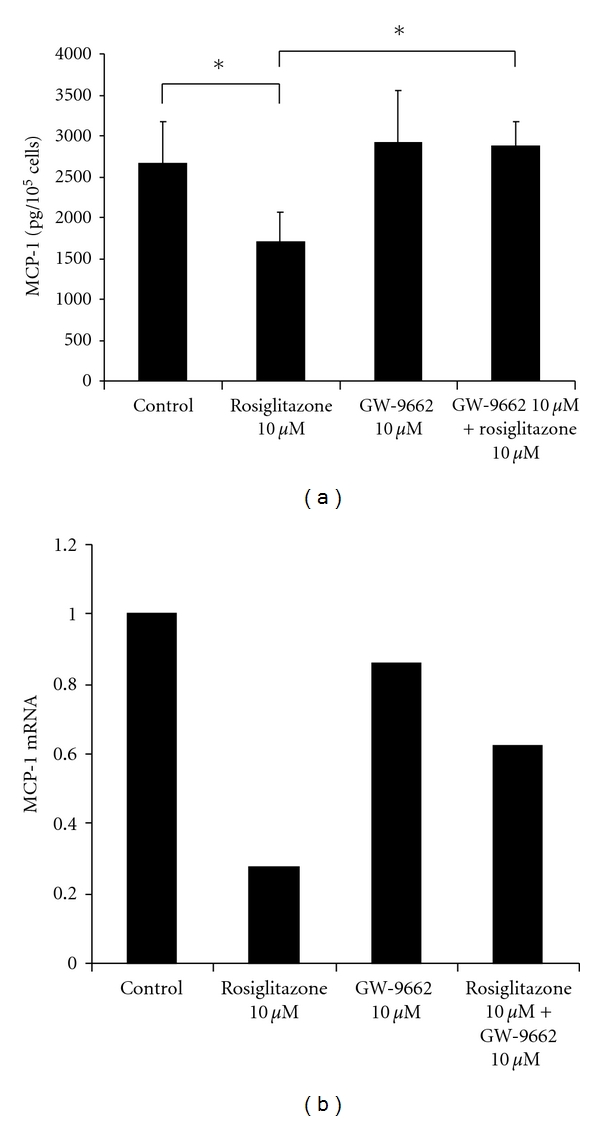
(a) PPAR-*γ*-dependency of the rosiglitazone effect on mesothelial MCP-1 release. MC were preincubated with GW-9662 10 *μ*M for 24 hours (or with control media) and then incubated with the given conditions for 8 hours. Cell supernatants were examined by ELISA. *indicates a *P* value < 0.05 (*n* = 5). (b) PPAR-*γ*-dependency of the rosiglitazone effect on mesothelial MCP-1 mRNA expression. MC were preincubated with GW-9662 10 *μ*M for 24 hours (or with control media) and then incubated with the given conditions for 8 hours. Total RNA was extracted and examined by RT-PCR. The figure shows a representative experiment out of three independent ones.

In order to investigate whether the effect of TZD on the mesothelial MCP-1 release is dependent on the TZD property to activate PPAR-*γ*, MC were preincubated with the PPAR-*γ* inhibitor GW-9662 (10 *μ*mol/L) or control media for 24 hours. Afterwards, cells were incubated with control media, rosiglitazone 10 *μ*mol/l, GW-9662 10 *μ*mol/L, or the combination of GW-9662 and rosiglitazone for 8 hours. In accordance with longer incubation times (Figure 2), rosiglitazone treatment led to a significant decrease in MCP-1 protein (1700 pg/10^5^ cells versus control: 2660 pg/10^5^ cells; *P* = 0.028). GW-9662 did not have an effect on MCP-1 secretion and resulted in similar MCP-1 protein levels compared to control. The blocking of PPAR-*γ* by administration of GW-9662 before a rosiglitazone application could completely prevent the rosiglitazone-induced attenuation in MCP-1 release (2870 pg/10^5^ cells versus 1700 pg/10^5^ cells; *P* = 0.039) (Figure 4(a)).

Corresponding results could be found on the transcriptional level: whilst rosiglitazone markedly reduced MCP-1 mRNA levels (0.28 versus 1), PPAR-*γ* blockade by GW-9662 resulted in considerably higher MCP-1 mRNA levels (0.62). GW-9662 alone had no effect on the MC MCP-1 mRNA production, as the administration resulted in a MCP-1 mRNA level comparable to the control (0.86) (Figure 4(b)).

### 3.5. Effect of Rosiglitazone on TNF*α*-Induced Enhanced Mesothelial MCP-1 Release

**Figure 5:**
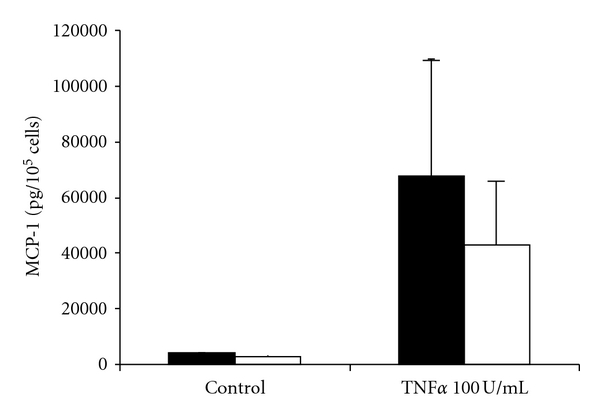
Effect of rosiglitazone on TNF*α*-induced enhancement of mesothelial MCP-1 secretion. MC were preincubated with rosiglitazone 10 *μ*M (white bars) or with control medium (black bars) for 24 hours. Afterwards, the culture media was replaced with media containing rosiglitazone 10 *μ*M (white bars) or not (black bars) to the given conditions. After an incubation time of 8 hours, culture supernatants were analysed by ELISA (*n* = 3).

The incubation of MC with TNF*α* (100 U/mL) resulted in a drastic increase in mesothelial MCP-1 secretion (67500 pg/10^5^ cells versus control 4000 pg/10^5^ cells). Prior incubation with rosiglitazone 10 *μ*mol/L and subsequent coincubation resulted in a one-third reduction in mesothelial MCP-1 levels (43000 pg/10^5^ cells versus 67500 pg/10^5^ cells) (Figure 5).

## 4. Discussion

MC are supposed to be critical in the pathogenesis of complications following PD treatment. By producing profibrotic [13, 14] and neoangiogenetic factors [15], they contribute to peritoneal fibrosis. In addition, MC are a major source of intraperitoneal MCP-1 and thus account for the recruitment of monocytes toward the peritoneal cavity [1]. Apart from an inflammatory reaction, this invasion contributes to peritoneal fibrosis by producing various cytokines and growth factors [2]. MCP-1 can be found in markedly elevated concentration in the dialysate of PD patients during and after episodes of peritonitis [16, 17]. IL-1*β*, IFN*γ*, and TNF*α* as well as high glucose concentrations (due to high osmolality and the polyol pathway) are found to increase the mesothelial MCP-1 synthesis rate [18, 19]. TZD are activators of PPAR-*γ*. This receptor is a member of the nuclear receptor family that includes 48 human transcription factors regulated by direct binding of steroid and thyroid hormones, vitamins, lipid metabolites, and xenobiotics [20]. By differential promotor usage and splicing two isoforms are generated: PPAR-*γ*1 which can be found on a variety of cell types and PPAR-*γ*2, which has an additional 30 amino acids at its N-terminal end and is expressed specifically in adipocytes [21]. We could now demonstrate the constitutive expression of PPAR-*γ*1 on human MC. In PD patients, there are some experiences with TZD: Lin et al. described that rosiglitazone improved glucose metabolism in nondiabetic uremic patients on CAPD [22], and Wong et al. found reduced insulin requirement and C-reactive protein levels in type 2 diabetic patients receiving peritoneal dialysis [23]. In the present study, we could demonstrate that TZD are able to reduce the constitutive MCP-1 release in MC by PPAR-*γ* stimulation. Furthermore, rosiglitazone was able to attenuate enhanced MCP-1 secretion resulting from a stimulation with the proinflammatory cytokine TNF*α*. Peng et al. found a decrease in high glucose concentration-induced mesothelial production of TGF*β*, collagen I, and fibronection secretion after treatment with troglitazone [14]. Some in vivo animal model studies point toward positive TZD effects on the peritoneal membrane in PD or peritoneal inflammation: Yao et al. found maintained peritoneal morphology and increased ultrafiltration after intraperitoneal administration of rosiglitazone in comparison to commercial PD solution alone [24]. Sandoval et al. described a reduction in the accumulation of AGEs as well as reduced fibrosis and angiogenesis resulting in an improved peritoneal function [25]. In accordance with our findings, Hornung et al. were able to demonstrate that the intraperitoneal administration of ciglitazone was able to significantly reduce the number of invading peritoneal macrophages following a thioglycollate-induced peritoneal inflammation [26]. PPAR-*γ* stimulation in MC may be a promising possibility in the attempt to minimize long-term PD complications. However, potential negative effects of the TZD (the only commercially available PPAR-*γ* activators at present) such as their ability to cause edema and their negative cardiovascular risk profile [27] should be considered critically.

## 5. Conclusions

PPAR-*γ*1 protein is expressed on human peritoneal MC. Its activation via rosiglitazone decreases the mesothelial release and mRNA expression of MCP-1 and attenuates the TNF*α*-induced enhancement in MCP-1 release in these cells. Therefore, the PPAR-*γ*1 receptor may be a therapeutic target to ameliorate peritoneal inflammation and long-time survival of the peritoneal membrane in PD. However, potential negative systemic effects of the TZD have to be considered critically.
